# RETRACTION: PD‐L1 Expressed From Tumor Cells Promotes Tumor Growth and Invasion in Lung Cancer via Modulating TGF‐β1/SMAD4 Expression

**DOI:** 10.1111/1759-7714.70390

**Published:** 2026-09-21

## Abstract

**RETRACTION**: ChenM.‐J. , WangY.‐C. , WangL. , ShenC.‐J. , ChenC.‐Y. and LeeH. , “PD‐L1 Expressed From Tumor Cells Promotes Tumor Growth and Invasion in Lung Cancer via Modulating TGF‐β1/SMAD4 Expression,” Thoracic Cancer 13, no. 9 (2022): 1322–1332, 10.1111/1759-7714.14388.PMC905831535373505

The above article, published online on 04 April 2022 in Wiley Online Library (wileyonlinelibrary.com), has been retracted by agreement between the journal Editor‐in‐Chief, Tateaki Naito; China Lung Oncology Group; and John Wiley & Sons Australia, Ltd. The retraction has been agreed upon following concerns raised by a third party. An investigation identified several duplications between images presented in Figures 1c and 1d; 1a and 1d; 3a and 3c; 3c and 3d; and 4c and 4d. The authors were contacted to comment on the concerns and provide supporting data but did not respond. The editors consider the results and conclusions to be unreliable. The authors did not respond to the retraction notice.

**RETRACTION**: M.‐J. Chen , Y.‐C. Wang , L. Wang , C.‐J. Shen , C.‐Y. Chen and H. Lee , “PD‐L1 Expressed From Tumor Cells Promotes Tumor Growth and Invasion in Lung Cancer via Modulating TGF‐β1/SMAD4 Expression,” Thoracic Cancer 13, no. 9 (2022): 1322–1332, 10.1111/1759-7714.14388.PMC905831535373505

The above article, published online on 04 April 2022 in Wiley Online Library (wileyonlinelibrary.com), has been retracted by agreement between the journal Editor‐in‐Chief, Tateaki Naito; China Lung Oncology Group; and John Wiley & Sons Australia, Ltd. The retraction has been agreed upon following concerns raised by a third party. An investigation identified several duplications between images presented in Figures 1c and 1d; 1a and 1d; 3a and 3c; 3c and 3d; and 4c and 4d. The authors were contacted to comment on the concerns and provide supporting data but did not respond. The editors consider the results and conclusions to be unreliable. The authors did not respond to the retraction notice.

